# Infection prevention and control practice for Crimean-Congo hemorrhagic fever—A multi-center cross-sectional survey in Eurasia

**DOI:** 10.1371/journal.pone.0182315

**Published:** 2017-09-08

**Authors:** Tom E. Fletcher, Abuova Gulzhan, Salih Ahmeti, Seif S. Al-Abri, Zahide Asik, Aynur Atilla, Nick J. Beeching, Heval Bilek, Ilkay Bozkurt, Iva Christova, Fazilet Duygu, Saban Esen, Arjun Khanna, Çiğdem Kader, Masoud Mardani, Faisal Mahmood, Nana Mamuchishvili, Natalia Pshenichnaya, Mustafa Sunbul, Tuğba Y. Yalcin, Hakan Leblebicioglu

**Affiliations:** 1 Liverpool School of Tropical Medicine, Liverpool, United Kingdom; 2 Department of Clinical Microbiology and Infectious Diseases, School of Medicine, Ondokuz Mayis University, Samsun, Turkey; 3 South-Kazakhstan State Pharmaceutical Academy, Shymkent, Republic of Kazakhstan; 4 Infectious Disease Clinic, University of Prishtina "Hasan Prishtina", Medical Faculty, Prishtina, Kosovo; 5 Infectious Diseases, Royal Hospital, Muscat, Oman; 6 Infectious Diseases and Clinical Microbiology, Tokat State Hospital, Tokat, Turkey; 7 Department of Infectious Diseases and Clinical Microbiology, Samsun Research and Training Hospital, Samsun, Turkey; 8 Department of Infectious Diseases, Siirt State Hospital, Siirt, Turkey; 9 Department of Microbiology, National Center of Infectious and Parasitic Diseases, Sofia, Bulgaria; 10 Department of Infectious Diseases, AY Ankara Oncology Research and Training Hospital, Ankara, Turkey; 11 Metro Centre for Respiratory Diseases, Metro Multispeciality Hospital, Noida, Uttar Pradesh, India; 12 Department of Infectious Diseases and Clinical Microbiology, Bozok University, Yozgat, Turkey; 13 Infectious Diseases Department, Faculty of Medicine, Shahid Beheshti University of Medical Sciences, Tehran, Iran; 14 Infectious Diseases, Department of Medicine, Aga Khan University Hospital, Karachi, Pakistan; 15 National Center for Disease Control and Public Health, Tbilisi, Georgia; 16 Department of Infectious Diseases, Rostov State Medical University, Rostov-on-Don, Russia; 17 Department of Infectious Diseases, Sivas Numune Hospital, Sivas, Turkey; National Institute of Health, ITALY

## Abstract

**Background:**

Crimean Congo Hemorrhagic Fever (CCHF) is a life threatening acute viral infection that presents significant risk of nosocomial transmission to healthcare workers.

**Aim:**

Evaluation of CCHF infection prevention and control (IP&C) practices in healthcare facilities that routinely manage CCHF cases in Eurasia.

**Methods:**

A cross-sectional CCHF IP&C survey was designed and distributed to CCHF centers in 10 endemic Eurasian countries in 2016.

**Results:**

Twenty-three responses were received from centers in Turkey, Pakistan, Russia, Georgia, Kosovo, Bulgaria, Oman, Iran, India and Kazakhstan. All units had dedicated isolation rooms for CCHF, with cohorting of confirmed cases in 15/23 centers and cohorting of suspect and confirmed cases in 9/23 centers. There was adequate personal protective equipment (PPE) in 22/23 facilities, with 21/23 facilities reporting routine use of PPE for CCHF patients. Adequate staffing levels to provide care reported in 14/23 locations. All centers reported having a high risk CCHFV nosocomial exposure in last five years, with 5 centers reporting more than 5 exposures. Education was provided annually in most centers (13/23), with additional training requested in PPE use (11/23), PPE donning/doffing (12/23), environmental disinfection (12/23) and waste management (14/23).

**Conclusions:**

Staff and patient safety must be improved and healthcare associated CCHF exposure and transmission eliminated. Improvements are recommended in isolation capacity in healthcare facilities, use of PPE and maintenance of adequate staffing levels. We recommend further audit of IP&C practice at individual units in endemic areas, as part of national quality assurance programs.

## Introduction

Crimean Congo Hemorrhagic Fever (CCHF) is a life threatening acute viral infection was first identified in 1944 in the Crimea, and later recognized as the cause of an outbreak in the Congo in 1969. It is geographically widespread across Africa, Eastern Europe, Asia and the Middle East [1]. Turkey is the current epicenter of CCHF activity, reporting up to 1000 confirmed cases annually [2], followed by Pakistan, Iran and Russia. The virus is transmitted to humans predominantly through tick bites, but also through exposure to blood and tissues of infected animals and humans [3]. It has a case fatality rate of 4–30% in hospitalized cases and there is no proven therapeutic or vaccine available [4]. Ribavirin has demonstrated in vitro activity against CCHFV and is used in some centers, but a meta-analysis of predominantly observational studies did not show any survival benefit.

Healthcare personnel are at risk from nosocomial infections [5] and the first such cases were described in Pakistan and later reported from many countries [6–11]. Severe CCHF with associated hemorrhage presents the highest risk, and failure to recognize the initial non-specific clinical features of CCHF remains a challenge both in endemic settings and in exported cases in travelers [5]. Pschenichnaya et al [7], recently reported a fatal case in Russia that resulted in nosocomial infection of eight healthcare workers (HCWs), whilst Conger et al [8], also highlighted the risk of exported cases, reporting a fatal case of CCHF in a US soldier evacuated to Germany resulting in nosocomial infection of two HCWs. Healthcare facilities can also act as amplifiers in outbreaks such as in Mauritania in 2003, when the index case directly infected a total of 15 HCWs, patients and visitors in the ward and emergency room setting, resulting in six deaths [12].

The route and circumstances of VHF nosocomial transmission are not always well-defined, but a recent multi-centre study from Turkey, clearly showed that needle stick injury and splash to mucous membranes were the commonest causes of high risk exposure in healthcare personnel. Fatal CCHF cases disproportionately generated more high risk exposures and a range of HCWs were affected, highlighting the requirement for broad educational programs, focused on sharps safety, personal protective equipment (PPE) use and early recognition of suspect cases. The data also suggested a possible post-exposure survival benefit of ribavirin [5](). Seroprevalence surveys of HCWs in endemic settings have demonstrated low rates of anti-CCHF IgG positivity overall [13,14], even when minimal PPE has been utilized. This further supports the consensus opinion that there is little human to human transmission of CCHFV especially in mild disease, and that this mostly occurs in nosocomial settings, with severe disease and well-defined high-risk exposures.

The nosocomial risk of other viral hemorrhagic fevers to healthcare workers was also clearly highlighted in the recent Ebola epidemic in West Africa, with significant numbers of HCWs infected [15]. International and national guidance exists detailing the infection, prevention and control precautions for VHF/CCHF [16], but nosocomial exposure continues to occur in endemic countries. This project was aimed at evaluating the CCHF IP&C practices in endemic countries by a cross-sectional survey in health care facilities that routinely manage CCHF cases. To our knowledge this is the first attempt to collectively survey and report CCHF IP&C practices in a range of endemic countries and to highlight training requirements.

## Materials and methods

**Fig 1:**
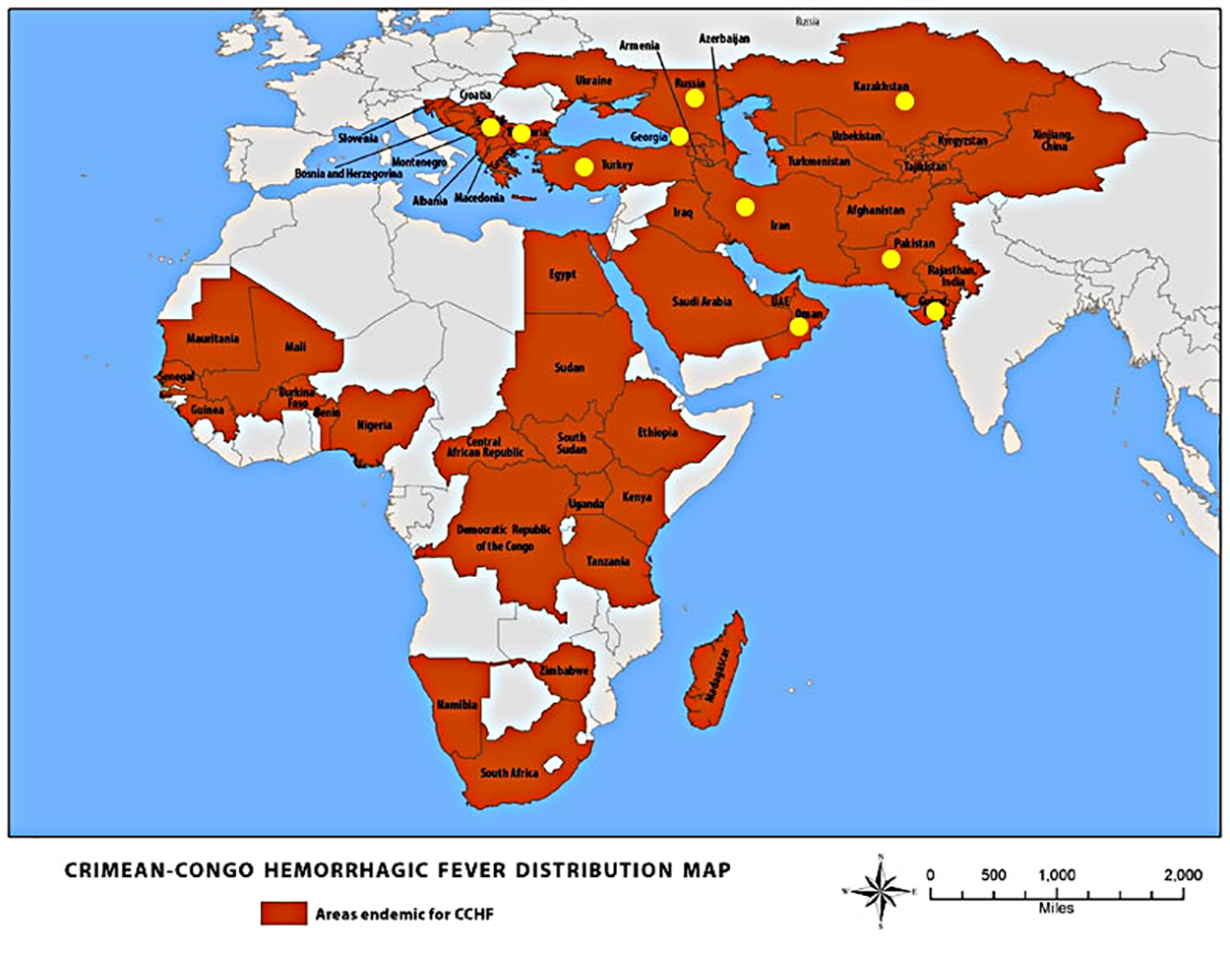
Crimean-Congo hemorrhagic fever distribution map with study sites (adapted with permission from centers for disease control and prevention).

A questionnaire was developed to gather information on CCHF infection prevention and control practices. Through an online platform contact points in the main Eurasian countries endemic for CCHF were invited to contribute (Fig 1). Respondents were senior physicians and identified through being: members of World Health Organization CCHF expert panels/national advisory boards; senior authors from the published literature from CCHF endemic countries; and as clinical experts working in major CCHF centers. The survey was designed and conducted as a service evaluation of CCHF IP&C practice in endemic countries and research and ethical approval was not required. All completed survey responses were received by April 2016 and respondents who had completed a response to at least one question were included for analysis. The questionnaire focused on isolation facilities, personal protective equipment use, infection control practice and educational/training requirements. It included a combination of closed-ended yes/no and multiple-choice questions, and open-ended questions for numerical answers (S1 File). We collected background data on the demographics of each hospital, national diagnostic support and CCHF guideline provision. The results were summarized in tabular form and analyzed utilizing descriptive statistical methods.

## Results

Twenty-three responses were received from centers that regularly manage CCHF in 10 countries. Fourteen responses were received from Turkey and one from CCHF centers in Pakistan, Russia, Georgia, Kosovo, Bulgaria, Oman, Iran, India and Kazakhstan. There was a CCHF National advisory board in 4/10 countries (Turkey, Iran, Georgia, Kosovo) and a CCHF National reference laboratory in 8/10 countries (Pakistan, Russia, Georgia, Kosovo, Bulgaria, Oman, Iran and Turkey). National CCHF guidelines existed in 7/10 countries and local hospital guidelines in 15/23 centers.

Most of the respondents were from Tertiary Healthcare facilities (18/23), all with Infectious Diseases specialty support. The bed capacity at each facility ranged from 20–2100 beds (median 646) and the median number of confirmed CCHF cases managed in the last 12 months was 8 (range 1–105, mean 20). The majority of the units (12/23) had managed fatal CCHF cases in the previous 12 months. The majority of units had a bio-safety level 2 laboratory (22/23), with 8/23 having access to a bio-safety level 3 laboratory. The time reported to receive a CCHF diagnosis by serology after sampling was a median 2 days (n = 21) (range 1–7 days). The time to get a CCHF diagnosis by PCR was a median 2 days (n = 22) (range 1–5 days) (Table 1).

**Table 1:** General characteristics of respondents (23 CCHF centers, 10 countries).

Characteristics	
	*Yes*, *number (%)*
CCHF National Advisory board	4/10 (40)
CCHF National Referral Laboratory	8/10 (80)
National CCHF Guidelines	7/10 (70)
Tertiary care facilities	18/23 (78.3)
Local CCHF Guidelines	15/23 (65.2)
Managed Fatal CCHF cases last 12 months	12/23 (52.1)
	*Median (range)*
Bed Capacity	646 (20–2100)
Total number of CCHF cases last 12 months	8 (1–105)

All units had dedicated isolation rooms for CCHF, with 17/23 also having designated isolation rooms in the Emergency Department (Table 2). In the infectious diseases department isolation rooms, there were anterooms in 10/23 (43%), dedicated ventilation systems in 8/23 (34.8%), negative pressure ventilation in 5/23 (21.7%) and High efficiency particulate air (HEPA) filtration in 4/23 (17.4%) centers. There was cohorting of confirmed cases in the same rooms in 15/23 centers and cohorting of suspect and confirmed cases in the same room in 9/23 centers. Relatives were allowed to enter the rooms of confirmed CCHF patients in 7/23 centers. There were dedicated HCWs to manage CCHF patients in 10/23 centers with adequate staffing levels to provide care reported in 14/23 locations, although 19/23 reported a reduction in nursing staff levels for CCHF in-patients overnight. Intensive care level (ICU) support to CCHF patients was available in all facilities (23/23), with dedicated separate ICU rooms available in 17/23 centers.

**Table 2:** CCHF infection prevention and control responses (23 centers).

Characteristics	*Yes*, *number (%)*
HCWs specifically allocated to CCHF patients	10/23 (43.5)
Adequate staffing to provide care to CCHF patients	14/23 (60.9)
Reduction in nursing staff levels for CCHF in-patients overnight	19/23 (82.6)
Isolation rooms for CCHF patients in the Emergency Department	17/23 (74)
Isolation rooms for CCHF patients in the Intensive Care Unit	17/23 (74)
Isolation rooms in the Infectious diseases have:	
- Anterooms	10/23 (43.5)
- Dedicated ventilation systems	8/23 (34.8)
- Negative pressure ventilation	5/23 (21.7)
- HEPA filtration	4/23 (17.4)
Cohorting of confirmed CCHF cases	16/23 (69.6)
Cohorting of suspect and confirmed CCHF cases together	9/23 (39.1)
Relatives allowed to enter CCHF patient rooms	7 /23 (30.4)
Adequate personal protective equipment (PPE) in the facility	22/23 (95.7)
Routine use of PPE when entering CCHF patient’s rooms	21/23 (91.3)
Adequate training in donning & doffing of PPE	20/23 (87)
Supervised donning & doffing of PPE	14/23 (60.9)
PPE donning & doffing posters available	18/23 (78.3)
Number of healthcare worker CCHF exposures in the last 5 years?	
- 1–5	18/23 (78.3)
- >5	5/23 (21.7)
Special burial protocol for fatal CCHF cases	18/23 (78.3)
Terminal cleaning of CCHF patient’s rooms	20/23 (87)
Needle safe devices used in CCHF patients	16/23 (69.6)
Frequency of Healthcare worker CCHF education:	
- Annually	13/21 (61.9)
- Monthly	1/21 (4.8)
- Once	(7/21) (33.3)

There were adequate levels and type of personal protective equipment (PPE) in 22/23 facilities. HCWs routinely utilized PPE on entering the rooms of patients with suspected/confirmed CCHF in 21/23 centers. Supervised donning and doffing of PPE was undertaken in 14/23 centers, with a PPE donning & doffing poster available in 18/23 centers. Five centers reported no supervision or posters to guide donning/doffing PPE. Needle safe devices were utilized in 16/23 centers and there a formal system for recording CCHF needle stick injuries (NSI) in 22/23 centers. All centers reported having a high risk CCHFV exposure (NSI or splash to mucous membranes) to a HCW occurring in last five years, with 18 centers reporting 1–5 exposures, and 5 centers reporting more than exposures (in Turkey and Pakistan). Terminal clean of isolation rooms occupied by CCHF patients is undertaken by 20/23 facilities, with the majority (18/23) having a special burial protocol for fatal cases.

Education about CCHF was provided to HCWs in 21/23 of the facilities. Education was provided annually in most centers (13/20), monthly in one center and once only in 7 centers. Respondents reported adequate provision of training in donning and doffing of personal protective equipment in 20/23 centers. Additional training requirements were requested in the following areas: Identification of potential cases 13/23; Isolation procedures 10/23; PPE use 11/23; PPE donning/doffing 12/23; Environmental disinfection 12/23; Disinfection of instruments 11/23; Waste management 14/23; Laboratory protocols 10/23; and prevention of HCW exposure 12/23 (Table 3).

**Table 3:** Additional IP&C training requirements requested by 23 CCHF units.

Training	*Number (%)*
Identification of potential cases	13/23 (56.5)
Isolation procedures	10/23 (43.5)
Personal protective equipment	11/23 (47.8)
Donning & doffing of personal protective equipment	12/23 (52.2)
Environmental/terminal cleaning	12/23 (52.2)
Medical equipment cleaning/disinfection	11/23 (47.8)
Waste Management	14/23 (60.9)
Laboratory protocols for managing CCHF samples	10/23 (43.5)
Prevention of HCW exposure CCHF	12/23 (52.2)

## Discussion

The protection of HCWs and patients from healthcare-related transmission of CCHF is equally as important as delivery of good clinical care, and underpinned by sound infection, prevention and control practice. This survey is the first to describe IP&C practice and educational priorities in centers with significant CCHF expertise in endemic countries in Eurasia. The centers that participated in the study manage more than 50% of the CCHF cases reported annually worldwide and are from a range of countries with different resources. The centers also have experience of managing the spectrum of CCHF disease severity with over 50% managing fatal cases, that present the highest nosocomial risk [5], in the last 12 months.

There is unquestionably a need to improve IP&C practice in endemic countries that is evidenced by all centers reporting high-risk exposures in the last 5 years, a quarter of which reported at least one high risk exposure annually. This is consistent with previous reports and regularly published cases series in the literature highlighting nosocomial outbreaks [17,18]. Key to HCW protection and prevention of nosocomial transmission is early recognition and diagnosis of CCHF cases. Molecular diagnosis through RT-PCR took a median of 2 days in the centers and can be improved through stream-lined processes and surveillance networks, supported by decentralization of diagnostic testing to endemic areas. In the future, this can be improved by access to simplified multiplex PCR platforms such as those used in the Ebola outbreak, including the Biofire Filmarray that provides a result in one hour. Equally as important as rapid diagnosis is heightened clinical suspicion and early risk assessment for VHF/CCHF, in both endemic areas and in travelers [19]. This allows early isolation of suspect cases, implementation of appropriate infection prevention and control precautions and reduces risk of nosocomial transmission. Previous series have highlighted that 25% of HCWs with high risk exposures to CCHFV came from patients not suspected as having CCHF [5]. Over 50% of centers in our study requested additional education with respect to identification and triage of CCHF, consistent with this need.

There was adequate provision of PPE in most centers, although this was not universally utilized by HCWs when entering patient rooms and the type and quality of PPE was not surveyed. One of the key lessons learnt from the West African Ebola outbreak is that the simple provision of adequate PPE is not enough to protect HCWs. Its use requires training, a safe environment and constant vigilance/quality assurance. Doffing of PPE is a recognized high risk activity, requires multiple steps and ideally should be guided by an external monitor and with visual aids. Supervised donning and doffing was undertaken in 14/23 centers with 18/23 utilizing posters as visual aids.

Poor compliance to the recommended PPE for CCHF has previously been reported. In 2012 PPE usage and exposures amongst HCWs and laboratory staff that manage CCHF patients and samples was investigated in a hospital in the hyperendemic region in Turkey, that has managed over 1200 confirmed cases [20]. Of 190 participants included PPE usage was reported to be 93.7% for gowns, 77.4% for gloves, and 38.9% for masks; with the highest compliance found in HCWs on the infectious diseases ward. Although there were relatively low rates of high risk exposures such as needle stick injuries, and only one CCHF IgG positive result, over 10% reported direct skin contact with blood. Higher seroprevalence rates in HCWs that manage CCHF cases has been shown in Iran [21]. Another study from Iran also reported that only 44% of HCWs wore gloves and masks during direct contact with CCHF patients, and 22% failed to observe any safety measure. HCWs with a history of percutaneous contact also had significantly lower knowledge scores about the disease [22].

Needle stick injuries are a clear risk for HCWs managing patients with VHF and daily venipuncture for CCHF patients is standard in many centers. Regular blood analysis helps recognize organ dysfunction, guide electrolyte replacement and most importantly directs blood component therapy, a key feature of CCHF case management. Use of needle safe devices should be universal, but were utilized in only 16/23 of centers, but reassuringly 22/23 had a system of recording NSIs. This is important both for subsequent root cause analysis and also consideration of post-exposure prophylaxis with ribavirin.

Protection of other patients and relatives from nosocomial transmission of CCHFV is equally as important as staff protection. To achieve this healthcare facilities require adequate isolation capacity from the emergency department through to definitive care by infectious diseases specialists and in intensive care units. Access to intensive care support was universal in all centers, but only 18/23 had dedicated isolation rooms in emergency departments and intensive care units. In endemic areas where high case-loads are seen cohorting of patients with confirmed CCHF is appropriate, and practically can be easier and safer for clinical staff. In endemic areas up to 50% of suspect CCHF cases are RT-PCR negative and it is hard to justify cohorting confirmed and suspect cases in the same room, as takes place in over one third of centers.

Although relatives and care-givers may have been exposed to CCHFV pre-admission, it is difficult to justify the risk of further exposure in a healthcare facility by allowing entry to patient’s rooms, as was reported by 7/23 centers. As CCHF progresses patients may become more infectious, and practical solutions can be found to allow relatives to visit without additional risk. Dedicated ventilation systems/HEPA filtration were only available in a minority of units, and although experts agree that there is no circumstantial or epidemiological evidence of an airborne transmission risk from VHF patients [23], a theoretical risk has been postulated [24]. Aerosol generating procedures, associated with managing critically ill CCHF patients present challenges, and although dedicated ventilation/filtration may not be required for all CCHF patients, capacity should be available, particularly in intensive care units. It is important however to maintain IP&C focus, training and resources on the main routes of VHF transmission through adherence to universal precautions, avoiding contact with a patient’s body fluids, minimizing contamination of the environment, and use of appropriate PPE that is fit for purpose and suitable for the person wearing it.

Addition IP&C training requirements were requested by the units in the areas detailed in Table 3 and should form the basis of future CCHF educational programs. These must be comprehensive, mandatory and include non-clinical staff and processes such as environmental cleaning, waste management, and review laboratory protocols for managing hazardous samples. Handling dead bodies or human remains of suspected or confirmed CCHF patients is a high-risk activity and although protocols exist in most units (18/23) bespoke training in this area must be delivered to mortuary/nursing staff. All training must be provided with consideration of susceptibility to human error and requires ongoing quality assurance and re-enforcement. Training was provided on an annual basis by most centers and when possible this should correlate with the onset of the CCHF season. Safe provision of clinical care to CCHF patients also requires adequate staffing levels, as well as appropriate equipment and a safe environment/processes. Clinical staff need to be highly trained and managing CCHF patients can be highly intensive, physically demanding and stressful. Recent nosocomial transmission of CCHF in Spain demonstrated that countries with low risk of CCHF should also train HCWs in CCHF IP&C and case management [25].

Only 14/23 centers reported adequate staffing levels to manage CCHF caseloads, with >75% reporting a reduction in nursing levels overnight. Adequate staffing levels is a key component of maintaining a safe environment and hospital/unit managers must make this a priority in line with their duty of care to staff and patients.

The main limitations of the study are that the majority of centers sampled are specialist tertiary referral centers for CCHF, and the results probably do not reflect general practice in endemic regions. It may be expected that IP&C standards would be higher in these centers, and the results probably underestimate broader deficiencies in primary /secondary healthcare facilities that frequently diagnose and initially manage CCHF cases. These are however, major centers representing locations where the majority of CCHF cases are managed worldwide and also manage more severe CCHF disease. There appears to be inclusion bias towards Turkish CCHF centers and this reflects the CCHF case load in Turkey and existing research networks. The other key limitation related to study design is that the IP&C practice was self-reported by the lead clinicians at the centers. Without independent additional assessment and audit of practice at these centers, that is beyond the scope of this study, it is hard to confirm accuracy of all the results. However, respondents were aware that the results would be anonymized and most of the key survey responses were objective measurements.

## Conclusions

This is the first study to evaluate IP&C practice in countries in Eurasia where CCHF is endemic. It provides evidence of ongoing nosocomial risk and highlights several areas where improvements in practice can be made. These include improvements in isolation capacity in healthcare facilities, particularly to reduce cohorting of suspect and confirmed case, use of personal protective equipment and maintaining adequate staffing levels. Educational priorities have been highlighted and we suggest further detailed audit of IP&C practice at individual units and endemic areas. Guidelines and regular education of HCWs are important, but must be supported by quality assurance that is overseen by respective Ministries of Health. Staff and patient safety must be improved and healthcare associated CCHF exposure and transmission eliminated.

## Supporting information

S1 File CCHF IP&C questionnaire. (DOCX)
