# Supplementary material for: Infection prevention and control practice for Crimean-Congo hemorrhagic fever—A multi-center cross-sectional survey in Eurasia
Source: PLoS One. 2017 Sep 8;12(9):e0182315. doi: 10.1371/journal.pone.0182315 (PMC5590734; doi:10.1371/journal.pone.0182315)
Supplement: S1 File — (DOCX) [file pone.0182315.s001.docx]

CCHF IP&C Questionnaire 2016

Name, City, Country

Do you have a CCHF National Advisory board/expert committee in your country? Yes/No

Do you have a CCHF National Reference Laboratory in your country ? Yes/No

What is the bed capacity in the hospital? ­­­­­­­­­­­­­­­­­­­______

What is the number of proven CCHF cases in that managed in the facility in 2015 ? ______

What is the number of fatal CCHF cases in the facility in 2015? ______

Is there an isolation room for CCHF patients in the emergency department? Yes/No

Are there dedicated rooms for CCHF patients in the facility/department/clinic Yes/No

In your department do the rooms have? (select from the following)

- Anterooms
- Dedicated ventilation
- Negative pressure ventilation
- HEPA filtration

What is the number of beds in the rooms? ______

Do you cohort confirmed cases with CCHF in the same room? Yes/No

Is there cohorting of suspected and confirmed cases in the same room? Yes/No

Are there dedicated HCWs for CCHF? Yes/No

Number of nurses in the clinic in the day time? ______

Number of nurses in the clinic on night shifts? ______

Do you allow relatives to enter the rooms of patients for care? Yes/No

In the Hospital Microbiology Laboratory is there a BSL2 present? Yes/No

Is there a BSL3 laboratory available? Yes/No

Is diagnosis of CCHF by ELISA? Yes/No

Average time to get CCHF ELISA results (days)? ______

Is there diagnosis of CCHF by PCR? Yes/No

Average time to get CCHF PCR results (days) ______

Adequate capability for preparation of fresh frozen plasma/apheresis platelets? Yes/No

Is Intensive care support available? Yes/No

Are there dedicated separate intensive care rooms? Yes/No

Is there routine use of PPE when entering the room of all CCHF patients? Yes/No

Is there adequate PPE in the facility? Yes/No

Is there supervised donning/doffing of PPE? Yes/No

Are donning/doffing guide posters utilized? Yes/No

Do you use needle-safe devices? Yes/No

Do you have an institutional recording system for needle stick injuries? Yes/No

How many HCW high risk exposures have you had in the last 5 years? ______

Do you have a special transfer service/protocol for patients with CCHF from one healthcare center to other in your country? Yes/No

Do you have a special burial protocol for patients died from CCHF in your hospital? Yes/No

Is there National guideline for CCHF? Yes/No

Are there Hospital guidelines/protocols/algorithms for CCHF? Yes/No

Is there adequate training on donning/doffing of PPE? Yes/No

How often is CCHF education to HCWs provided? ______

Do you have a terminal cleaning procedure for the room after discharge of CCHF cases? Yes/No

Indicate the topics that your facility needs additional training:

Identification of potential cases

Isolation procedures

Personal protective equipment

Donning & doffing of personal protective equipment

Environmental/terminal cleaning

Medical equipment cleaning/disinfection

Waste Management

Laboratory protocols for managing CCHF samples

Prevention of HCW exposure CCHF
